# Within-Host Multiplication and Speed of Colonization as Infection Traits Associated with Plant Virus Vertical Transmission

**DOI:** 10.1128/JVI.01078-19

**Published:** 2019-11-13

**Authors:** Alberto Cobos, Nuria Montes, Marisa López-Herranz, Miriam Gil-Valle, Israel Pagán

**Affiliations:** a Centro de Biotecnología y Genómica de Plantas (UPM-INIA), Universidad Politécnica de Madrid, Pozuelo de Alarcón, Madrid, Spain; b E.T.S.I. Agronómica, Alimentaria y de Biosistemas, Campus de Montegancedo, Universidad Politécnica de Madrid, Pozuelo de Alarcón, Madrid, Spain; University of Maryland, College Park

**Keywords:** *Arabidopsis thaliana*, cucumber mosaic virus, seed transmission, turnip mosaic virus, vertical transmission, virus multiplication, within-host movement, virulence

## Abstract

Although vertical transmission from parents to offspring through seeds is an important fitness component of many plant viruses, very little is known about the factors affecting this process. Viruses reach the seed by direct invasion of the embryo and/or by infection of the ovules or the pollen. Thus, it can be expected that the efficiency of seed transmission would be determined by (i) virus within-host multiplication and movement, (ii) the ability of the virus to invade gametic tissues, (iii) plant seed production upon infection, and (iv) seed survival in the presence of the virus. However, these predictions have seldom been experimentally tested. To address this question, we challenged 18 Arabidopsis thaliana accessions with *Turnip mosaic virus* and *Cucumber mosaic virus*. Using these plant-virus interactions, we analyzed the relationship between the effect of virus infection on rosette and inflorescence weights; short-, medium-, and long-term seed survival; virulence; the number of seeds produced per plant; virus within-host speed of movement; virus accumulation in the rosette and inflorescence; and efficiency of seed transmission measured as a percentage and as the total number of infected seeds. Our results indicate that the best estimators of percent seed transmission are the within-host speed of movement and multiplication in the inflorescence. Together with these two infection traits, virulence and the number of seeds produced per infected plant were also associated with the number of infected seeds. Our results provide support for theoretical predictions and contribute to an understanding of the determinants of a process central to plant-virus interactions.

**IMPORTANCE** One of the major factors contributing to plant virus long-distance dispersal is the global trade of seeds. This is because more than 25% of plant viruses can infect seeds, which are the main mode of germplasm exchange/storage, and start new epidemics in areas where they were not previously present. Despite the relevance of this process for virus epidemiology and disease emergence, the infection traits associated with the efficiency of virus seed transmission are largely unknown. Using turnip mosaic and cucumber mosaic viruses and their natural host Arabidopsis thaliana as model systems, we have identified the within-host speed of virus colonization and multiplication in the reproductive structures as the main determinants of the efficiency of seed transmission. These results contribute to shedding light on the mechanisms by which plant viruses disperse and optimize their fitness and may help in the design of more-efficient strategies to prevent seed infection.

## Abstract (precis)

One of the major factors contributing to plant virus long-distance dispersal is the global trade of seeds. This is because more than 25% of plant viruses can infect seeds, which are the main mode of germplasm exchange/storage, and start new epidemics in areas where they were not previously present. Despite the relevance of this process for virus epidemiology and disease emergence, the infection traits associated with the efficiency of virus seed transmission are largely unknown. Using turnip mosaic and cucumber mosaic viruses and their natural host Arabidopsis thaliana as model systems, we have identified the within-host speed of virus colonization and multiplication in the reproductive structures as the main determinants of the efficiency of seed transmission. These results contribute to shedding light on the mechanisms by which plant viruses disperse and optimize their fitness and may help in the design of more-efficient strategies to prevent seed infection.

## INTRODUCTION

The ability to be transmitted is arguably the most important determinant of parasite fitness. Indeed, most theoretical models of the evolution of parasites consider infection traits, such as virulence, i.e., the effect of infection on host fitness (1), or within-host multiplication, as relevant factors for parasite fitness because they affect the efficiency of between-host transmission (2–4). Parasites can be transmitted from host to host, for instance, by vectors or by contact (i.e., horizontal transmission) and/or from parents to offspring (i.e., vertical transmission) (3, 5). Most effort devoted to understanding parasite transmission has focused on identifying ecological and genetic determinants of horizontal transmission (6, 7), and comparatively much less is known about the determinants of vertical transmission. However, a wide range of human, animal, and plant parasites that are causal agents of severe diseases are vertically transmitted or have both horizontal and vertical transmission (8, 9). Hence, vertical transmission is a major component of parasite fitness, and exploring the factors involved in its efficiency is central to understanding host-parasite interactions (3, 10).

Vertical transmission is particularly frequent in plant viruses, as more than 25% of all known species are vertically transmitted through seeds (11, 12). Seed transmission is highly relevant for plant virus epidemiology (11–13). Seed infection provides the virus with the means to persist for long periods of time (years) when hosts and/or vectors are not available, as many seed-transmitted viruses can survive within the seed as long as it remains viable (11, 12). This facilitates virus emergence and reemergence in plant populations (5, 14). Seed transmission also allows for long-distance dissemination of the virus (14, 15). Indeed, evidence shows that bird dispersion and human trade of infected seeds have allowed cross-continental jumps of some plant viruses (16, 17). Finally, seed transmission represents an important source of primary inoculum for many viruses with this mode of transmission, which are disseminated afterwards via insect vectors. In this way, plant viruses initiate damaging epidemics even at very low seed transmission rates (11, 18, 19). Although the central role of vertical transmission in plant virus epidemiology is widely acknowledged, very little is known regarding which host and virus traits interact to determine the efficiency of seed transmission (11, 14).

In general, plant viruses achieve seed transmission in two ways according to the distribution of the virus in the seed. The first is through contamination of the seed coat. In this case, during germination, the virus infects the seedling through abrasions caused by soil particles (11, 20). This mechanism of seed transmission has been reported for a few viruses and is relatively well understood only for tobamoviruses (21). The second, and most common, method of virus seed transmission is through invasion of the seed embryo (11, 22). Embryo invasion may occur in two non-mutually exclusive ways: indirectly by infection of plant gametes prior to fertilization, either the ovules or the pollen, or directly from the mother plant to the embryonic tissue after fertilization (23). Hence, for seed transmission to occur, it is crucial that the virus reaches and invades plant reproductive organs before gametogenesis and/or while the embryo is still accessible from mother cells, without affecting gamete/embryo viability. Plant defense responses that regulate virus virulence may enhance or prevent embryo invasion, for instance, by altering the virus distribution in the plant, which may modify the efficiency of seed transmission (24). Thus, it has been proposed that the efficiency of seed transmission would be determined by (i) the ability of the virus to reach gametic tissues, which would be determined by the speed of within-host movement; (ii) the ability of the virus to invade gametic tissues, which would be associated with virus multiplication in reproductive organs; (iii) plant progeny production upon infection (i.e., virus virulence); and (iv) gamete and embryo survival in the presence of the virus (3, 10, 11, 20, 23).

Experimental evidence of the role of these infection traits in virus seed transmission is scarce. Plant genes involved in the efficiency of virus seed transmission have been identified only in soybean (24). In this host, *Soybean mosaic virus* seed transmission is controlled by plant gene homologs of Arabidopsis thaliana *DCL3* and *RDR6*, which are involved in small RNA-mediated gene silencing (24). Similarly, virus seed transmission determinants have been analyzed in only a few species. Genetic variation in *Barley stripe mosaic virus* (BSMV), *Cucumber mosaic virus* (CMV), and *Pea seed-borne mosaic virus* genes encoding the replicase and movement proteins has been associated with the efficiency of seed transmission (25, 26). This would be compatible with a role of virus multiplication, virulence, and movement. However, whether (and how) infection traits affect the efficiency of seed transmission has been seldom analyzed, and with contradictory results. Pagán et al. (27) showed that reduced CMV virulence and within-host multiplication were associated with an increased efficiency of seed transmission in Arabidopsis thaliana. Stewart et al. (28) also reported a negative correlation between BSMV virulence and the efficiency of virus seed transmission in barley, but no link to virus multiplication was detected. These works studied the effect of infection traits on the efficiency of seed transmission using univariate analyses. However, during infection, virus multiplication, movement, and reduction of plant fitness occur simultaneously, such that the efficiency of seed transmission would be determined by their combined (and not necessarily equally important) effects (20). To date, such multivariate effects have not been analyzed, and the infection traits associated with virus seed transmission (and their relative importance) are still poorly understood (14).

The efficiency of seed transmission may also affect parasite evolution. The fitness of vertically transmitted parasites is highly dependent on host reproductive potential, as hosts need to reproduce for the parasite to infect new individuals. Accordingly, the “continuum hypothesis” proposes that parasites with a higher efficiency of vertical transmission will evolve toward lower virulence and, because virulence is an unavoidable consequence of parasite growth, toward lower within-host multiplication (3, 29, 30). These predictions have seldom been experimentally tested, particularly for plant viruses (5, 27). Moreover, these works commonly estimated the efficiency of vertical transmission by determining the proportion of offspring that carry the parasite. This measure of the efficiency of seed transmission does not account for variation in the number of propagules that different host genotypes can produce, which may affect parasite fitness. Indeed, it has been proposed that the total number of infected progeny reflects more accurately the contribution of vertical transmission to parasite fitness and therefore is more directly linked to parasite evolution (2, 3, 5, 10, 31). According to this theory, parasite vertical transmission-related fitness is the result of the interplay between the percentage of infected progeny, virulence, and the amount of progeny produced by the host. The first determines the proportion of infected progeny, the second determines the reduction of the host’s maximal progeny production, and the third determines progeny production upon infection (2, 3, 5, 31). Thus, understanding how the efficiency of seed transmission affects virus evolution requires considering both the percentage of seed transmission and the total number of infected propagules.

To address these questions, we utilized *Turnip mosaic virus* (TuMV) (*Potyviridae*), CMV (*Bromoviridae*), and Arabidopsis thaliana (Brassicaceae) (referred to here as *Arabidopsis*). Both viruses are commonly found in natural populations of *Arabidopsis* at a prevalence of up to 80% (32), indicating that *Arabidopsis*-TuMV and *Arabidopsis*-CMV interactions are significant in nature. In *Arabidopsis*, CMV has been shown to be seed transmitted (14, 27), and the high TuMV prevalence in natural *Arabidopsis* populations early in the spring (before aphid flights) suggests that this virus may also be seed transmitted (32, 33). In addition, TuMV and CMV infections in *Arabidopsis* differ in traits proposed to be associated with the efficiency of seed transmission. For instance, TuMV multiplies at lower levels than CMV, whereas TuMV is more virulent (34; N. Montes, V. Vijayan, and I Pagán, submitted for publication). Also, both viruses differentially affect the survival of infected seeds (35). Thus, our experimental system allows testing of theoretical predictions on the host and virus traits that determine seed transmission under different infection conditions. To do so, in six *Arabidopsis* accessions, we measured (i) TuMV and CMV speed of within-host movement and multiplication, as proxies of the ability of the virus to reach and invade gametic tissues, respectively; (ii) the effect of virus infection on plant growth and progeny production, as a measure of virulence; and (iii) short-, medium-, and long-term survival of infected seeds. Using multivariate mixed models, we investigated which of these infection traits are associated with the efficiency of virus seed transmission, quantified both as a percentage and as the total number of infected seeds, and their relative importance. We constructed global models that considered data for both viruses together as well as virus-specific models, such that we could differentiate traits broadly associated with seed transmission from virus species-specific determinants of this process. We validated the resulting models by measuring, in a larger set of 18 *Arabidopsis* accessions, seed transmission and the most relevant estimators of these traits, and we compared the values predicted by our models with experimental quantifications of seed transmission.

## RESULTS

### TuMV and CMV seed transmission in *Arabidopsis*.

**FIG 1:**
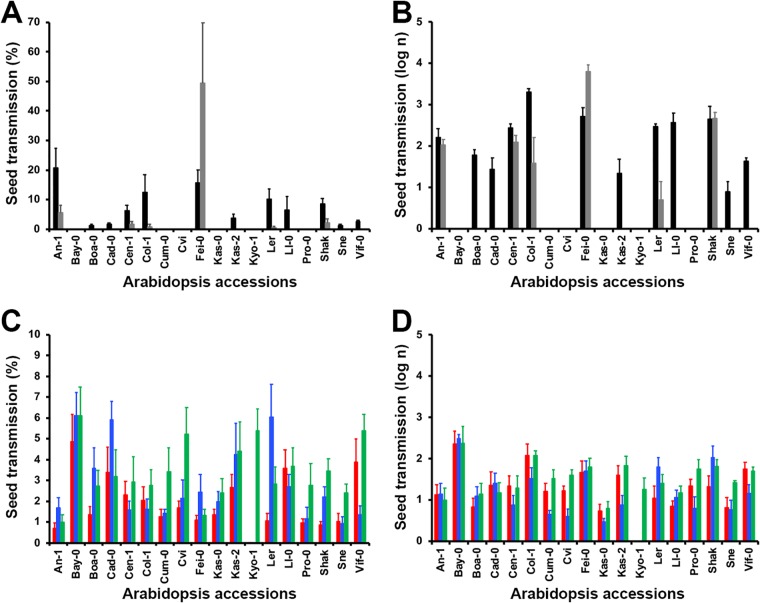
Virus seed transmission in *Arabidopsis*. TuMV seed transmission percentage (A) and log number of infected seeds (B) and CMV seed transmission percentage (C) and log number of infected seeds (D) in 18 *Arabidopsis* accessions are shown. Data for JPN1-TuMV (black), UK1-TuMV (gray), De72-CMV (red), Fny-CMV (blue), and LS-CMV (green) are represented. Note the different scale of each panel.

We quantified seed transmission as a percentage (*ST*) and as the total number (*IS*) of TuMV- and CMV-infected seeds in 18 *Arabidopsis* accessions (Fig. 1 and Table 1). In TuMV-infected plants, the virus was detected in 12/18 accessions, whereas seeds harboring CMV were detected in all accessions. On average, the percentage of infected seeds was higher in TuMV-infected (4.17% ± 1.53%) than in CMV-infected (2.60% ± 0.23%) plants (Wald χ^2^_1,775_ = 8.59 [where subscript 1 is the degree of freedom of chi squared and 775 the degrees of freedom of the error term in the same chi-squared analysis]; *P* = 0.003). Similar results were obtained when seed transmission was quantified as the number of infected seeds (2.13 ± 0.19 versus 1.35 ± 0.07 for TuMV and CMV, respectively) (Wald χ^2^_1,775_ = 13.45; *P* < 0.001). Both measures of seed transmission depended on the *Arabidopsis* accession (Wald χ^2^_17,759_ ≥ 206.42; *P* < 0.001) and on the interaction between accession and virus species (Wald χ^2^_17,759_ ≥ 199.62; *P* < 0.001). Thus, we analyzed both *ST* and *IS* for each virus separately.

**TABLE 1:** *Arabidopsis* accessions used in this work and their geographical origin and life cycle

Accession	Origin	Life cycle^*a*^
An-1	Amberes (Belgium)	Short
Bay-0	Bayreuth (Germany)	Short
Boa-0	Boadilla (Spain)	Long
Cad-0	Candelario (Spain)	Long
Cen-1	Centenera (Spain)	Short
Col-1	Columbia (unknown)	Short
Cum-0	Cumbres Mayores (Spain)	Long
Cvi	Cape Verde Islands	Short
Fei-0	Santa María da Feira (Portugal)	Short
Kas-0	Kashmir (India)	Long
Kas-2	Kashmir (India)	Long
Kyo-1	Kyoto (Japan)	Long
Ler	Landsberg (Poland)	Short
Ll-0	Llagostera (Spain)	Long
Pro-0	Proaza (Spain)	Short
Shak	Shakdara (Tajikistan)	Short
Sne	Sierra Nevada (Spain)	Short
Vif-0	Villafáfila (Spain)	Short

a Length of the accession life cycle as defined previously (50).

TuMV isolate UK1 (UK1-TuMV) was seed transmitted in 6/18 accessions, and JPN1-TuMV was transmitted in 12/18 (Fig. 1A and B), with the percentage of TuMV seed transmission varying according to the *Arabidopsis* accession (Wald χ^2^_17,306_ = 230.64; *P* < 0.001) but not to the virus isolate (UK1-TuMV, 3.34% ± 2.73%; JPN1-TuMV, 4.99 ± 1.46) (Wald χ^2^_1,306_ = 2.51; *P* = 0.113). The interaction between these factors was significant (Wald χ^2^_17,306_ = 91.76; *P* ≤ 0.001). Similar trends were observed when the number of TuMV-infected seeds was analyzed (Fig. 1A and B).

The three CMV isolates were seed transmitted in all *Arabidopsis* accessions (Fig. 1C and D). The percentages of CMV seed transmission differed between *Arabidopsis* accessions (Wald χ^2^_17,419_ = 81.59; *P* < 0.001) and between virus isolates (Wald χ^2^_2,419_ = 22.38; *P* < 0.001), with similar values for Fny-CMV (2.59% ± 0.46%) and LS-CMV (2.37% ± 0.34%) and lower values for De72-CMV (1.83% ± 0.30%). The interaction between these factors was significant (Wald χ^2^_17,419_ = 56.49; *P* = 0.009). Again, similar results were obtained when CMV seed transmission was quantified as the number of infected seeds (Fig. 1C and D).

These results indicated that the efficiency of seed transmission depended on the host-virus genotype-per-genotype interaction. Thus, infection traits under the control of the host and/or the virus may modulate this process. We subsequently quantified, in different *Arabidopsis* accessions inoculated with TuMV and CMV, a set of infection-related traits, and we analyzed their relationship with the efficiency of seed transmission.

### TuMV and CMV infection in *Arabidopsis*.

Six out of the 18 *Arabidopsis* accessions (An-1, Bay-0, Cad-0, Cum-0, Ll-0, and Fei-0) and 2 virus isolates (Fny-CMV and JPN1-TuMV) were selected to analyze the relationship between 9 traits proposed to be linked to virus seed transmission and *ST* and *IS*. Specifically, we calculated the effects of virus infection on rosette weight (*RW*) (ratio of *RW* of infected plants/*RW* of mock-inoculated plants [*RW_i_*/*RW_m_*]), inflorescence weight (*IW*) (*IW_i_*/*IW_m_*), and short-term (seed germination percentage at time zero [*G*_0_]) (*G*_0_*_i_*/*G*_0_*_m_*), medium-term (*G*_24_*_i_*/*G*_24_*_m_*), and long-term (*G*_48_*_i_*/*G*_48_*_m_*) seed survival; virulence (*V*); the number of seeds produced per infected plant (*SN_i_*); virus within-host speed of movement (*SM*); and virus accumulation in rosette (*VA_R_*) and inflorescence (*VA_I_*) leaves, plus *ST* and *IS* (see Materials and Methods).

Overall, the effect of infection on *RW*, *IW*, and *G*_24_ plus *V*, *SN_i_*, *SM*, *VA_R_*, and *VA_I_* differed according to the virus isolate (Wald χ^2^_1,100_ ≥ 3.70; *P* ≤ 0.054), the *Arabidopsis* accession (Wald χ2_5,100_ ≥ 11.94, *P* ≤ 0.036), and the accession-per-isolate interaction (Wald χ^2^_5,100_ ≥ 22.83; *P* < 0.001), whereas the effects of virus infection on *G*_0_ and *G_48_* did not (Wald χ^2^ ≤ 8.97; *P* ≥ 0.110) (Table 2). Note that differences in *SM* according to the *Arabidopsis* accession and the accession-per-isolate interaction could not be analyzed, as this trait was quantified as an accession-specific, rather than as an individual-specific, trait (see Materials and Methods). These results prompted us to analyze the variation in these infection traits for each virus separately. In JPN1-TuMV-infected plants, the effect of infection on *RW*, *IW*, and *G*_24_ plus *V*, *SN_i_*, *VA_R_*, and *VA_I_* varied according to the *Arabidopsis* accession (Wald χ^2^_5,49_ ≥ 17.38; *P* ≤ 0.002), and the effects of infection on *G*_0_ and *G*_48_ did not (Wald χ^2^_5,49_ ≤ 8.41; *P* ≥ 0.078) (Table 2). Similar results were obtained for Fny-CMV-infected plants (Wald χ^2^_5,46_ ≥ 19.79; *P* < 0.001), except that in this case, only the effect of virus infection on *G*_0_ did not vary between accessions (Wald χ^2^_5,46_ = 4.56; *P* = 0.472) (Table 2). Note that Fny-CMV virulence was negative in some accessions, indicating plant overcompensation for the effect of infection on its fitness, as previously reported (33, 34; Montes et al., submitted).

**TABLE 2:** Virus infection parameters measured in the six *Arabidopsis* accessions utilized to construct multivariate models for virus seed transmission

Virus and parameter^*a*^	Mean value for accession ± SE						
	An-1	Bay-0	Fei-0	Cad-0	Cum-0^*b*^	Ll-0	Avg^*c*^
JPN1-TuMV							
*ST*	22.38 ± 6.50	0.00 ± 0.00	20.27 ± 5.82	1.06 ± 0.67	0.00 ± 0.00	6.08 ± 0.70	8.24 ± 1.85
*IS*^*d*^	2.33 ± 0.33	0.00 ± 0.00	2.25 ± 0.53	1.15 ± 0.53	0.00 ± 0.00	1.83 ± 0.31	1.25 ± 0.18
*RW_i_*/*RW_m_*	0.83 ± 0.23	0.98 ± 0.13	0.46 ± 0.02	0.75 ± 0.03	0.30 ± 0.02	0.03 ± 0.00	0.56 ± 0.07
*IW_i_*/*IW_m_*	0.55 ± 0.07	0.67 ± 0.10	0.68 ± 0.05	0.76 ± 0.03	ND	0.16 ± 0.02	0.55 ± 0.04
*V*	0.76 ± 0.09	0.67 ± 0.09	0.55 ± 0.16	0.25 ± 0.05	1.00 ± 0.00	0.90 ± 0.01	0.72 ± 0.04
*SN_i_*^*d*^	3.19 ± 0.27	3.20 ± 0.36	3.75 ± 0.19	4.36 ± 0.03	0.00 ± 0.00	2.57 ± 0.12	2.94 ± 0.21
*SM*	1.09 ± 0.00	0.87 ± 0.00	1.40 ± 0.00	0.60 ± 0.00	ND	0.92 ± 0.00	1.00 ± 0.03
*VA_R_*	2.59 ± 0.37	0.58 ± 0.07	0.49 ± 0.29	0.48 ± 0.04	2.82 ± 0.21	1.94 ± 0.57	1.54 ± 0.18
*VA_I_*	0.69 ± 0.16	0.08 ± 0.02	0.33 ± 0.06	0.16 ± 0.04	ND	0.08 ± 0.04	0.28 ± 0.05
*G*_0_*_i_*/*G*_0_*_m_*	0.99 ± 0.01	0.98 ± 0.01	0.99 ± 0.00	1.01 ± 0.01	ND	1.00 ± 0.01	0.99 ± 0.00
*G*_24_*_i_*/*G*_24_*_m_*	0.95 ± 0.01	1.01 ± 0.02	0.92 ± 0.12	1.05 ± 0.01	ND	0.99 ± 0.03	0.98 ± 0.01
*G*_48_*_i_*/*G*_48_*_m_*	0.94 ± 0.08	1.02 ± 0.12	1.05 ± 0.18	1.08 ± 0.15	ND	0.94 ± 0.29	1.00 ± 0.07

Fny-CMV							
*ST*	1.54 ± 0.82	1.20 ± 0.23	1.78 ± 0.50	2.58 ± 0.43	6.17 ± 1.49	6.85 ± 1.39	3.27 ± 0.47
*IS*^*d*^	1.15 ± 0.41	1.27 ± 0.20	1.32 ± 0.34	2.69 ± 0.47	2.42 ± 0.10	3.31 ± 0.13	2.01 ± 0.15
*RW_i_*/*RW_m_*	0.54 ± 0.07	0.38 ± 0.02	0.34 ± 0.02	0.11 ± 0.02	0.17 ± 0.03	0.40 ± 0.04	0.32 ± 0.02
*IW_i_*/*IW_m_*	0.57 ± 0.07	0.34 ± 0.03	0.30 ± 0.04	0.27 ± 0.05	0.17 ± 0.04	0.93 ± 0.07	0.42 ± 0.04
*V*	0.50 ± 0.07	0.60 ± 0.03	0.74 ± 0.04	0.05 ± 0.16	−0.65 ± 0.25	−0.57 ± 0.05	0.13 ± 0.09
*SN_i_*^*d*^	3.84 ± 0.06	3.63 ± 0.04	3.65 ± 0.09	4.63 ± 0.06	4.36 ± 0.10	4.86 ± 0.02	4.13 ± 0.07
*SM*	0.42 ± 0.00	0.47 ± 0.00	0.57 ± 0.00	0.71 ± 0.00	0.93 ± 0.00	0.75 ± 0.00	0.64 ± 0.02
*VA_R_*	1.48 ± 0.14	3.07 ± 0.63	2.00 ± 0.16	2.44 ± 0.29	2.64 ± 0.30	1.39 ± 0.07	2.19 ± 0.16
*VA_I_*	0.25 ± 0.10	0.21 ± 0.07	0.23 ± 0.07	0.32 ± 0.06	0.28 ± 0.13	0.41 ± 0.08	0.28 ± 0.04
*G*_0_*_i_*/*G*_0_*_m_*	1.00 ± 0.01	1.00 ± 0.01	1.00 ± 0.00	1.00 ± 0.00	1.00 ± 0.00	1.01 ± 0.01	1.00 ± 0.00
*G*_24_*_i_*/*G*_24_*_m_*	1.00 ± 0.02	0.97 ± 0.02	1.00 ± 0.02	0.74 ± 0.12	0.79 ± 0.04	0.81 ± 0.08	0.89 ± 0.03
*G*_48_*_i_*/*G*_48_*_m_*	0.84 ± 0.15	0.98 ± 0.06	0.88 ± 0.18	0.81 ± 0.18	0.81 ± 0.09	0.61 ± 0.11	0.82 ± 0.06

a *SM* values have a standard error of 0.00, as they were measured as an *Arabidopsis* accession-specific trait.

b Cum-0 plants infected by JPN1-TuMV did not produce inflorescence and seeds, and the associated parameters could not be determined (ND).

c Average value for each parameter across all six *Arabidopsis* accessions.

d Log values are shown.

The seed transmission percentage and number of infected seeds were also estimated in the six *Arabidopsis* accessions. In this subset of accessions, *ST* and *IS* significantly varied according to the virus isolate (Wald χ^2^_1,100_ ≥ 6.25; *P* ≤ 0.012), the *Arabidopsis* accession (Wald χ^2^_5,100_ ≥ 39.29; *P* < 0.001), and the interaction between them (Wald χ^2^_5,100_ ≥ 56.46; *P* < 0.001). In agreement, virus-specific analyses showed that for both viruses, *ST* and *IS* varied significantly between *Arabidopsis* accessions (Wald χ^2^ ≥ 30.62; *P* < 0.001). *ST* and *IS* in the six *Arabidopsis* accessions showed similar trends between experiments (compare data in Fig. 1 and Table 2). Indeed, bivariate tests indicated a significant association between values in the two experiments for both *ST* and *IS* (*R*^2^ ≥ 0.49; *P* < 0.001).

Thus, all the analyzed infection traits, except for the effect of infection on short-term seed survival, showed variability according to the virus isolate, the *Arabidopsis* accession, and/or both factors.

### Association between CMV and TuMV seed transmission and viral infection traits in *Arabidopsis*.

Since the efficiency of seed transmission depended on the plant-virus genotype-per-genotype interaction, and we showed a similar variation for most infection traits proposed to be associated with this mode of transmission, we considered these infection traits as potential estimators of seed transmission. Thus, we performed more-detailed analyses of the association between infection traits and virus seed transmission utilizing multiple-regression model selection analyses (Table 3).

**TABLE 3:** Model selection analyses for TuMV and CMV percentages and total numbers of infected seeds^*g*^

Model structure^*a*^	*R*^2^*_c_*^*b*^	Log likelihood	AIC^*c*^	Δ*_i_*^*d*^	ω*_i_*^*e*^
*ST* (%)					
G, −4.55 + 10.30 · *VA_I_* (53) + 3.75 · *SM* (41) − 1.81 · *IW_i_*/*IW_m_* (2) − 4.19 · *V* (1) + 2.10 · *VA_R_* (1) + 4.80 · *G*_0_*_i_*/*G*_0_*_m_* (1) − 2.88 · *G*_24_*_i_*/G_24_*_m_* (1)	0.91*	−226.43	472.86	2	0.48
T, −13.59 + 1.49 · *VA_I_* (53) + 4.58 · *SM* (24) − 1.98 · *V* (14) − 1.49 · *IW_i_*/*IW_m_* (6) + 12.62 · *G*_0_*_i_*/*G*_0_*_m_* (2) − 0.88 · *G*_24_*_i_*/G_24_*_m_* (1)	0.99*	−101.73	219.45	8	0.20
C, −1.13 + 1.09 · *VA_I_* (37) + 3.19 · *SM* (37) − 0.75 · *G*_48_*_i_*/*G*_48_*_m_* (18) + 0.60 · *IW_i_*/*IW_m_* (8)	0.71*	−85.14	182.27	5	0.33

*IS* (log *n*)^*f*^					
G, 0.95 + 0.06 · *ST* (41) + 0.24 · *SN_i_* (23) − 0.59 · *V* (20) + 0.18 · *VA_I_* (10) − 0.42 · *RW_i_*/*RW_m_* (4) − 0.39 · *G*_48_*_i_*/*G*_48_*_m_* (2)	0.78*	−74.49	168.98	6	0.25
T, −2.59 + 0.05 · *ST* (45) + 0.97*SN_i_* (20) − 1.18 · *V* (15) + 1.17 · *SM* (7) + 0.21 · *VA_I_* (7) − 1.35 · *IW_i_*/*IW_m_* (4) − 0.48 · *G*_48_*_i_*/*G*_48_*_m_* (2)	0.81*	−32.01	86.23	9	0.18
C, 0.45 + 0.12 · *ST* (25) − 0.36 · *V* (24) + 1.90 · 10^−5^ · *SN_i_* (20) + 1.90 · *SM* (13) 0.11 · *VA_I_* (10) − 1.22 · *RW_i_*/*RW_m_* (4) + 1.08 · *IW_i_*/*IW_m_* (4)	0.78*	−39.37	96.74	15	0.13

a The relative importance (percent) of each estimator variable is shown in parentheses. G, global model; T, TuMV-specific model; C, CMV-specific model.

b Conditional correlation coefficient. Asterisks indicate significant correlations (*P* < 0.01).

c AIC, Akaike’s information criterion.

d Number of models closely competing with the best-ranked model (Δ*_i_* of <2 out of 511 for *ST* and 2,047 for *IS* models tested). Δ*_i_* is the difference between the AIC of a given model and that of the best-ranked model and quantifies how models compete (for the best-ranked model, Δ*_i_* = 0; for substantial empirical support, Δ*_i_* = 1 to 2; for considerably less support, Δ*_i_* = 2 to 7; for no support, Δ*_i_* > 10) (68).

e AIC model weight as ω*_i_* = exp(−0.5Δ*_i_*)/Σexp(−0.5Δ*_i_*). The larger the ω value, the greater the likelihood of the model relative to the competing models. The maximum ω*_i_* is 1.

f The number of infected seeds (*IS*) was normalized using a logarithmic transformation, and the resulting values were used for model construction.

g Model structures for *ST* included the effect of virus infection on rosette (*RW_i_*/*RW_m_*) and inflorescence (*IW_i_*/*IW_m_*) weights and on short-term (*G*_0_*_i_*/*G*_0_*_m_*), medium-term (*G*_24_*_i_*/*G*_24_*_m_*), and long-term (*G*_48_*_i_*/*G*_48_*_m_*) seed survival; virulence (*V*); virus within-host speed of movement (*SM*); and virus accumulation in rosette (*VA_R_*) and inflorescence (*VA_I_*) leaves. Model structures for *IS* also included *ST* and the total log number of seeds produced per plant (*SN*). Best-ranked models are shown.

**FIG 2:**
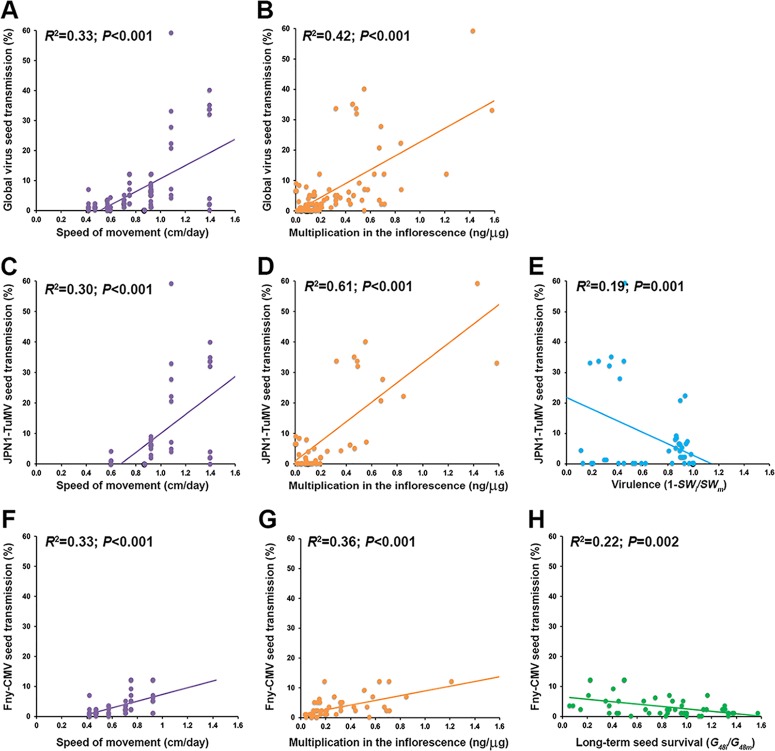
Bivariate relationships between percent virus seed transmission and infection traits. Regressions considering data for both viruses together (A and B), only JPN1-TuMV (C to E), and only Fny-CMV (F to H) are shown. Linear relationships of percent virus seed transmission and speed of virus movement in centimeters per day (purple), virus multiplication in the inflorescence in nanograms of viral RNA per microgram of total RNA (orange), virulence as 1 − (*SW_i_/SW_m_*) (light blue), and long-term seed survival as *G*_48_*_i_*/*G*_48_*_m_* (green) are represented.

In order to identify infection traits explaining virus seed transmission at large, we first constructed global multivariate models by merging the data from plants infected by both TuMV and CMV (see Materials and Methods). The best-ranked model contained *SM*, *V*, *VA_R_*, *VA_I_*, and the effect of infection on *IW*, *G*_0_, and *G*_24_ (*R*^2^ = 0.91; *P* < 0.001). *VA_I_* and *SM* had the highest relative importance (53% and 41%, respectively), explaining most of the variance in *ST* (Table 3). Because additional and/or different infection traits could determine seed transmission in a virus species-specific manner, we also constructed multivariate models for data on seed transmission for each virus separately. The model best explaining JPN1-TuMV *ST* included *SM*, *VA_I_*, *V*, and the effect of infection on *IW*, *G*_0_, and *G*_24_ (*R*^2^ = 0.99; *P* < 0.001). Again, *VA_I_* and *SM* were the most important estimators (relative importance, 53% and 24%, respectively), with virulence playing a less relevant role (relative importance, 14%) (Table 3). In addition, the best-ranked model explaining Fny-CMV *ST* included *SM*, *VA_I_*, and the effect of infection on *IW* and *G*_48_ (*R*^2^ = 0.71; *P* < 0.001), with *SM* and *VA_I_* being the most important estimators (both having a relative importance of 37%) and *G*_48_*_i_*/*G*_48_*_m_* having lower relative importance (18%). Thus, our results indicated that *VA_I_* and *SM* were chief estimators of *ST*, with *V* and *G*_48_*_i_*/*G*_48_*_m_* playing secondary roles in a virus-specific manner (Table 3). Regardless of the utilized data set, bivariate analyses indicated a positive association between *ST* and the main infection traits identified in our multivariate models: *SM* (*R*^2^ ≥ 0.30; *P* < 0.001) and *VA_I_* (*R*^2^ ≥ 0.37; *P* < 0.001) (Fig. 2). A weaker but still significant negative relationship between *ST* and the virus-specific secondary estimators (*V* and *G*_48_*_i_*/*G*_48_*_m_*) was also detected (*R*^2^ ≥ 0.19; *P* ≤ 0.002) (Fig. 2).

**FIG 3:**
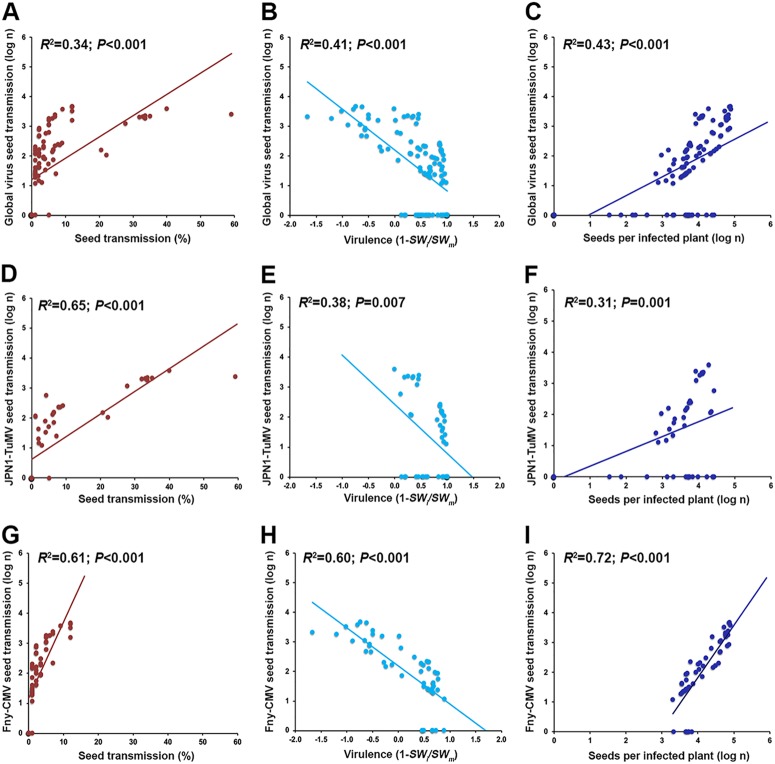
Bivariate relationships between the number of virus-infected seeds and infection traits. Regressions considering data for both viruses together (A to C), only JPN1-TuMV (D to F), and only Fny-CMV (G to I) are shown. Linear relationships of the log number of virus-infected seeds and percent virus seed transmission (brown), virulence as 1 − (*SW_i_/SW_m_*) (light blue), virus multiplication in the inflorescence in nanograms of viral RNA per microgram of total RNA (orange), and log number of seeds per infected plant (dark blue) are represented.

**FIG 4:**
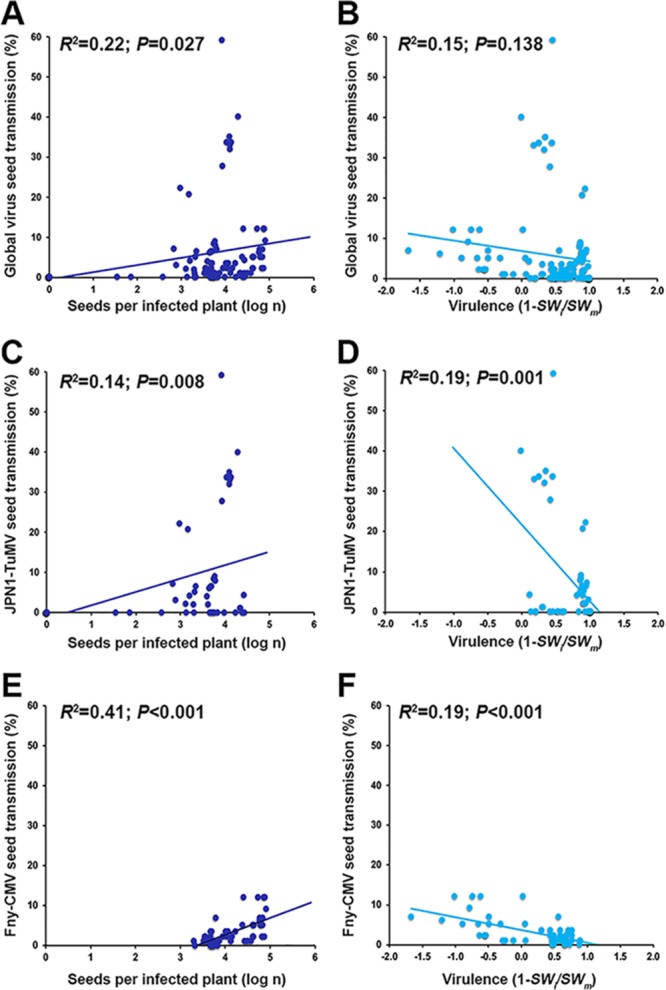
Bivariate relationships between the main predictors of the number of virus-infected seeds. Regressions considering data for both viruses together (A and B), only JPN1-TuMV (C and D), and only Fny-CMV (E and F) are shown. Linear relationships of percent infected seeds and log number of seeds per infected plant (dark blue) and of percent infected seeds and virulence as 1 − (*SW_i_*/*SW_m_*) (light blue) are represented.

The best-ranked global model explaining *IS* contained *ST*, *SN_i_*, *V*, *VA_I_*, and the effect of infection on *RW* and *G*_48_ (*R*^2^ = 0.78; *P* < 0.001). In this case, *ST*, *SN_i_*, and *V* had the highest relative importance (41%, 23%, and 20%, respectively) (Table 3). The best model explaining JPN1-TuMV *IS* included the same estimators as the ones for the best global model but replacing *RW_i_*/*RW_m_* by *IW_i_*/*IW_m_* and adding *SM* (*R*^2^ = 0.81; *P* < 0.001). Again, *ST*, *SN_i_*, and *V* were the most important estimators (relative importance, 45%, 20%, and 15%, respectively). The best-ranked model explaining Fny-CMV *IS* included *ST*, *V*, *SN_i_*, *SM*, *VA_I_*, and the effect of infection on *RW* and *IW* (*R*^2^ = 0.78; *P* < 0.001), with *ST*, *V*, and *SN_i_* being the most important estimators (relative importance, 25%, 24%, and 20%, respectively) (Table 3). Bivariate analyses indicated that *IS* was positively associated with *ST* (*R*^2^ ≥ 0.34; *P* < 0.001) and with *SN_i_* (*R*^2^ ≥ 0.31; *P* < 0.001) and negatively associated with *V* (*R*^2^ ≥ 0.38; *P* ≤ 0.001) (Fig. 3). These results suggested that *ST* in general could be negatively associated with virus virulence and positively associated with the progeny production of infected plants, expanding the information provided by the *ST*-explaining models. Thus, we analyzed the association between these traits (Fig. 4). *ST* was positively associated with *SN_i_* when both viruses were considered either together or separately (*R*^2^ ≥ 0.14; *P* ≤ 0.027). In agreement with *ST*-explaining models, a weak negative association between virulence and *ST* was detected in the virus-specific models (*R*^2^ ≥ 0.19; *P* < 0.001) but not in the global model (*R*^2^ = 0.15; *P* = 0.138) (Fig. 4).

### Model accuracy.

**FIG 5:**
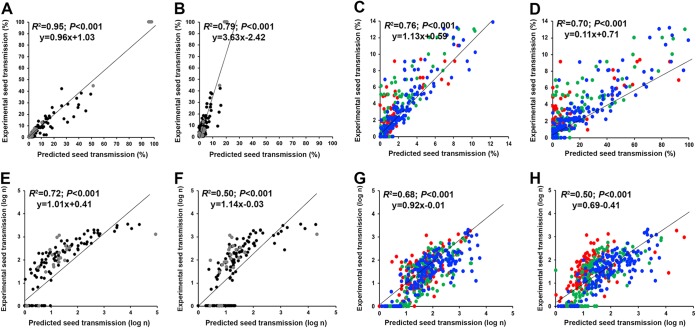
Association between experimental and estimated virus seed transmission of 5 TuMV and CMV isolates in 18 *Arabidopsis* accessions. Correlations between estimated values of percent TuMV seed transmission derived from the TuMV-specific model (A) and from the global model (B), percent CMV seed transmission derived from the CMV-specific model (C) and from the global model (D), the log number of TuMV-infected seeds derived from the TuMV-specific models (E) and from the global model (F), and the log number of CMV-infected seeds derived from the CMV-specific models (G) and from the global model (H) and the corresponding experimental values are shown. Data for UK1-TuMV (gray), JPN1-TuMV (black), LS-CMV (green), Fny-CMV (blue), and De72-CMV (red) are represented. Note the different scale of each panel.

It could be argued that our multivariate models are based on data from six *Arabidopsis* accessions and one isolate per virus inoculated at a given host phenological stage, and therefore, the derived results are specific only for these particular plant-virus genotype-per-genotype interactions and inoculation conditions. We analyzed the applicability of our models to other *Arabidopsis* accessions and TuMV/CMV isolates by using them to estimate the efficiency of seed transmission in the set of 18 *Arabidopsis* accessions and 5 virus isolates inoculated at the same phenological stage as the 6 accessions used for model construction. To do so, we quantified the relevant infection traits identified by the models (see Data Set S1 in the supplemental material), and we incorporated these values into the best-ranked global and virus-specific models to predict percentages and numbers of virus-infected seeds. The resulting values were compared with those obtained by experimental testing of seed infection (Fig. 5).

**FIG 6:**
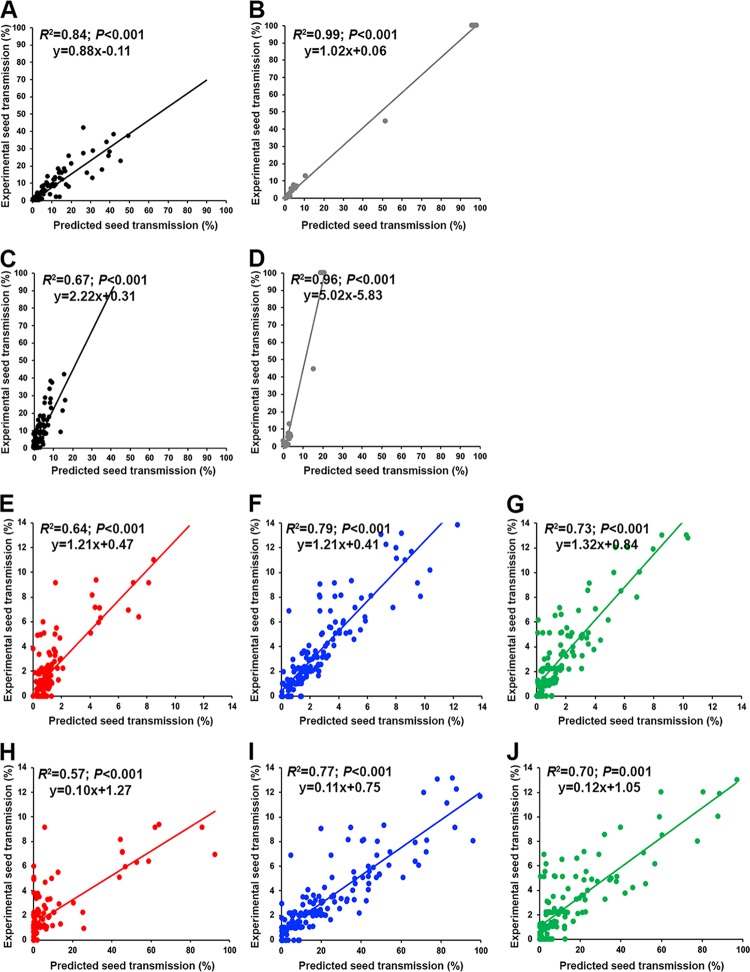
Association between experimental and estimated virus seed transmission of the 2 TuMV and 3 CMV isolates in 18 *Arabidopsis* accessions. (A to D) Correlations of percent TuMV seed transmission derived from the TuMV-specific model (A and B) and from the global model (C and D) to the corresponding experimental values. (E to J) Correlations of percent CMV seed transmission derived from the CMV-specific model (E to G) and from the global model (H to J) to the corresponding experimental values. Data for JPN1-TuMV (black), UK1-TuMV (gray), LS-CMV (green), Fny-CMV (blue), and De72-CMV (red) are represented.

**FIG 7:**
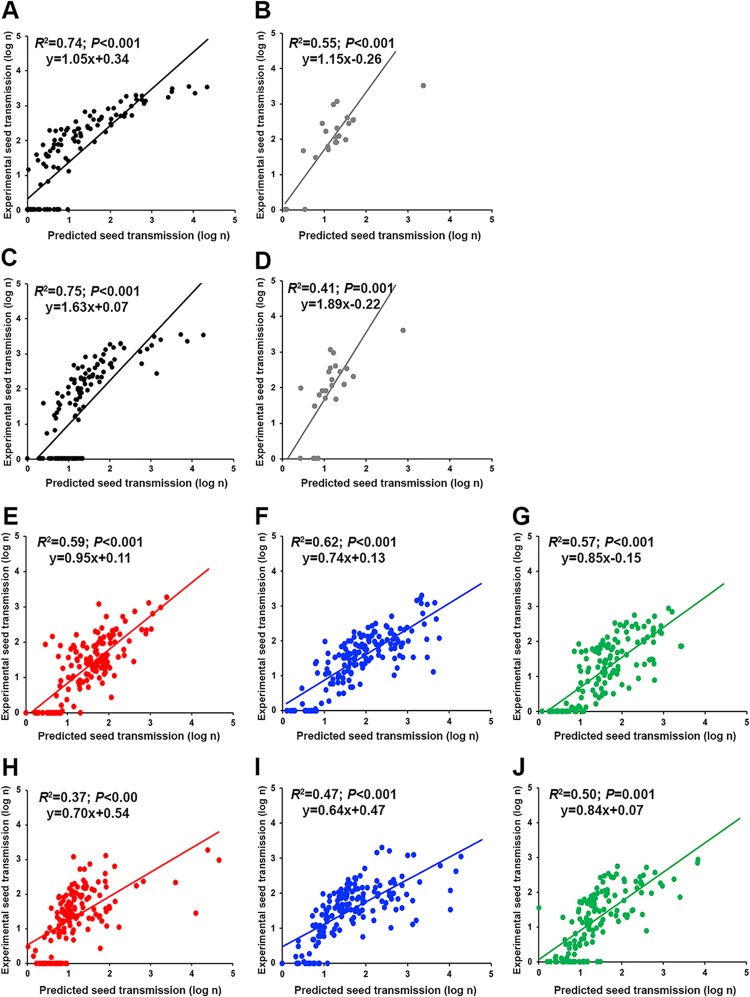
Association between experimental and estimated virus seed transmission of the 2 TuMV and 3 CMV isolates in 18 *Arabidopsis* accessions. (A to D) Correlations of the number of TuMV-infected seeds derived from the TuMV-specific model (A and B) and from the global model (C and D) to the corresponding experimental values. (E to J) Correlations of the number of CMV-infected seeds derived from the CMV-specific model (E to G) and from the global model (H to J) to the corresponding experimental values. Data for JPN1-TuMV (black), UK1-TuMV (gray), LS-CMV (green), Fny-CMV (blue), and De72-CMV (red) are represented.

Bivariate analyses of the relationship between TuMV and CMV experimental and predicted percentages of seed transmission indicated a significant positive correlation between both variables using either the global model or the virus-specific ones (*R*^2^ ≥ 0.70; *P* < 0.001) (Fig. 5A to D). However, prediction accuracy depended on the model. The equations of the linear correlations indicated that virus-specific models accurately predicted percentages of CMV and TuMV seed transmission (*x* coefficients near 1) (Fig. 5A and C). Similar results were obtained when predic tions for each isolate were analyzed separately (Fig. 6). On the other hand, the global model underestimated TuMV *ST* (*x* coefficient = 3.63) and overestimated CMV *ST* (*x* coefficient = 0.10), particularly at seed transmission values of >10% (Fig. 5B and D). Virus isolate-specific comparisons yielded similar biases (Fig. 6). Experimental and predicted numbers of infected seeds per plant were also significantly correlated for both global and virus-specific models (*R*^2^ ≥ 0.50; *P* < 0.001), although *R*^2^ values were generally lower than those for percent seed transmission (Fig. 5E to H). Again, virus-specific models performed better (*x* coefficient near 1) than the global one, but under- and overestimates of TuMV and CMV *IS*, respectively, were smaller than in the case of *ST*-estimating models (Fig. 5). Note that prediction of the absence of infected seeds was poorer in *IS* than in *ST* models. Similar results were obtained when data for each virus isolate were analyzed separately (Fig. 7).

Hence, for the host phenological stage at inoculation used in our experiments, our multivariate models fairly estimate trends of TuMV and CMV seed transmission across *Arabidopsis* accessions, i.e., high versus low seed transmission, and virus-specific models infer seed transmission values more accurately than the global ones.

## DISCUSSION

The ability to be transmitted is a key component of parasite fitness (2, 3, 5, 9, 10). Although vertical transmission from parents to offspring is not infrequent among plant parasites and plays a central role in their epidemiology, little is known about the mechanistic basis of this mode of transmission, particularly in plant viruses (14, 36). Here, we tested the hypothesis that virus transmission through seeds is associated with (i) the ability of the virus to move from the entry points to the plant reproductive structures, (ii) the ability of the virus to invade these reproductive structures, (iii) the capacity of the infected plant to produce progeny, and (iv) the capacity of infected progeny to survive (20, 36).

The efficiency of TuMV and CMV seed transmission depended on the *Arabidopsis* accession-virus species/isolate interaction, indicating that traits controlled by the host and/or the parasite determine this process. Global multivariate models identified the virus speed of within-host movement and its level of multiplication in the plant inflorescence as the best estimators of percent seed transmission. These variables can be considered proxies for the ability of the virus to reach and invade gametic tissues, respectively (11). Bivariate analyses indicated that the faster the virus spread across the reproductive structures, the higher its efficiency of seed transmission. Seed transmission requires reaching the plant reproductive structures during a particular window of time that in general is quite narrow: the embryo is accessible after fertilization and before the suspensor’s programmed cell death, and pollen and ovule invasion must occur prior to fertilization (11, 23). These periods last only a few days in *Arabidopsis* (37, 38). Hence, faster within-host movement would allow the virus to reach the reproductive organs within the required time frame in a larger number of flowers/siliques. This is compatible with experimental analyses showing that, in general, earlier virus infection leads to a higher efficiency of seed transmission (11, 14). Reaching the reproductive organs at the right moment does not guarantee seed transmission, as the virus also needs to gain entry into the seed (20). It has been proposed that a higher level virus multiplication in the plant reproductive structures favors embryo/gametophyte invasion by promoting virus crossing of the boundary between the maternal and progeny tissues (11, 39). In line with this prediction, our results indicate that a higher level of virus multiplication in the inflorescence increases the percentage of infected seeds. This observation also agrees with previous work showing that a higher percentage of seed transmission correlates with high virus titers in flowers, ovules, and/or pollen (19, 26, 40). Moreover, the only host genes identified as genetic determinants of plant virus seed transmission are involved in small RNA-mediated gene silencing, which modulates virus multiplication (24). Note that in our experiments, plants were inoculated at an early developmental stage. Virus inoculation of older plants would reduce the number of flowers/siliques that the virus can reach during the appropriate window of time, especially for the most basal ones (14, 20). In this context, the speed of within-host movement would likely become critical for seed transmission, increasing its relative importance in the multivariate models.

Virus-specific multivariate models confirmed that the speed of within-host movement and the level of multiplication in the inflorescence were major determinants of virus seed transmission. Interestingly, these models identified other traits as minor estimators of virus vertical transmission, which differed between TuMV and CMV. For TuMV, the combination of the two above-mentioned factors and virus virulence explained 91% of the variation in percent seed transmission. TuMV is regarded as a sterilizing parasite because it frequently prevents seed production in a number of *Arabidopsis* accessions, in which no vertical transmission is attained (41, 42). Hence, the identification of TuMV virulence as being negatively associated with seed transmission efficiency likely reflects that it determines the presence/absence of vertical transmission rather than having a role in the mechanism of this process. For CMV, the third trait (negatively) associated with the efficiency of vertical transmission was the long-term survival of infected seeds. Note that seed aging is posterior to our measures of the efficiency of vertical transmission. Thus, a lower long-term seed survival rate is likely a consequence of infection and suggests that the presence of the virus in the seed reduces its viability. We did not determine whether the seeds dying during the 48-h accelerated-aging treatment were mostly those harboring the virus. However, bivariate analyses using averaged values of the six accessions indicated that the percentage of CMV seed transmission explained 66% of the variance in the percentage of dead aged seeds. Hence, our results suggest that long-term seed survival could be a modifier of the efficiency of seed transmission in the long run. Alternatively, a lower long-term survival rate of seeds from infected plants could reflect maternal effects, but we did not find a significant difference in the weights of single seeds between infected and mock-inoculated plants, which argues against this possibility. The negative relationship between the efficiency of CMV seed transmission and long-term seed survival is in apparent contradiction with experimental analyses showing that infection of *Arabidopsis* plants by Fny-CMV renders seeds with improved tolerance to deterioration (35). However, those authors used Col-0 and a 24-h accelerated-aging treatment, an accession not included in our work and conditions for which we showed that the effect of Fny-CMV infection on seed survival significantly varies between accessions.

It is worth noting that we identified the same determinants of percent vertical transmission in two virus species with very different life histories and outcomes of infection. This suggests that our results would be applicable to the prediction of virus seed transmission in other host-virus interactions. Indeed, although some of the parameters used for model validation were estimates, both global and virus-specific ones predicted trends of seed transmission (i.e., higher versus lower transmission rates) in other *Arabidopsis* accessions and TuMV/CMV isolates with medium to high accuracy. More-accurate prediction of seed transmission values required the use of virus-specific models, which indicates that fine-tuning of the model estimative power needs to include virus-specific secondary determinants of seed transmission. Whether our models are applicable to other viruses or host species should be explored further. In any case, at least for TuMV and CMV, our results will help map the host and virus genes controlling the infection traits associated with the efficiency of seed transmission. This will allow the identification of candidate genetic determinants of this process, which currently remain elusive.

More than one-quarter of all known plant viruses have been reported to be vertically transmitted (11, 12), and this is likely an underestimate, as every year, more species, either long known or newly discovered, are described to be transmitted through seeds. CMV vertical transmission has been extensively reported (14, 43), whereas to date, TuMV has been considered strictly horizontally transmitted through insect vectors (11, 44). Our results indicate that in *Arabidopsis*, TuMV is transmitted through seeds and in some accessions with high efficiency, adding this to the list of vertically transmitted plant viruses. As mentioned above, in *Arabidopsis*, TuMV is considered a sterilizing virus (41, 42). Host sterilization allows host resources to be diverted from reproduction to survival, which increases the infectious period and the level of parasite multiplication. These modifications maximize horizontal transmission but at the cost of no vertical one (45–47). Hence, host sterilization is thought to be selectively advantageous for parasites that have strict horizontal transmission. However, our results show that TuMV maintains a mixed mode of transmission. Similarly, the fungus Atkinsonella hypoxylon has both modes of transmission in species of the genus *Danthonia* despite inducing plant sterility (48, 49). In this pathosystem, the coexistence of vertical and horizontal transmission has been explained on the basis of a vertical transmission-virulence trade-off. That is, in plants with most flowers sterilized, the few that are fertile produce a higher proportion of infected seeds than plants with fewer sterilized flowers (48, 49). Thus, higher virulence favors vertical transmission. This is not the case for TuMV, as the most virulent isolate (UK1-TuMV) had a seed transmission efficiency similar to that of a less virulent one (JPN1-TuMV). More importantly, the percentage of JPN1-TuMV seed transmission was negatively correlated with virulence and positively correlated with the number of seeds produced per infected plant, indicating no vertical transmission-virulence trade-off (Fig. 4). On the other hand, tolerance to TuMV infection may explain why seed transmission is maintained in the virus population: One-third of the 18 *Arabidopsis* accessions analyzed here avoided UK1-TuMV sterilization, and in all of them, the virus was seed transmitted. In these accessions, tolerance is attained by shortening the host growth period, which triggers plant reproduction before the plant experiences the full cost of infection (Montes et al., submitted). This reduces the resources available for virus multiplication, which in turn prevents maximization of horizontal transmission (45). Thus, TuMV seed transmission may compensate for the loss of virus fitness due to suboptimal horizontal transmission in tolerant accessions.

Our global and virus species-specific models explaining the total number of infected seeds, which best reflects the contribution of vertical transmission to virus fitness (2, 3, 5, 9, 10), support the link between virulence/tolerance and seed transmission. Indeed, these models identified virulence as one of the most important estimators, which was negatively associated with the number of infected seeds per plant. These results are therefore compatible with theoretical elaborations on parasite evolution under mixed modes of transmission (see the introduction).

Multivariate models also detected percent of seed transmission and the number of seeds produced by infected plants as the main estimators of the number of infected seeds, with both estimators being positively associated with this trait. The six *Arabidopsis* accessions utilized to build multivariate models were selected to represent plants with short and long life cycles (Table 1). In the absence of infection, short-lived accessions generally produce more seeds than long-lived ones (50, 51). Upon infection by JPN1-TuMV, these short-lived accessions showed a smaller reduction in seed production and a higher percentage of seed transmission than long-lived ones, which would explain the positive association between the number of infected seeds and the three main estimators of this trait. On the other hand, the efficiency of Fny-CMV seed transmission was higher in long-lived accessions. These accessions are known to display higher tolerance to CMV than short-lived ones (50, 51). Under our conditions, this allowed long-lived accessions to produce a larger number of seeds upon CMV infection than short-lived ones (Table 2). As a consequence, again, a higher level of seed production upon Fny-CMV infection was positively associated with the percentage of seed transmission and, by extension, with the number of infected seeds. Hence, the role of percent seed transmission and the number of seeds produced by infected plants as major estimators of the total number of infected seeds can be explained as a combination of plant allometry and tolerance to virus infection.

Not surprisingly, models generally included the speed of within-host movement of the virus and its multiplication in the inflorescence as secondary determinants of the number of infected seeds, the two traits associated with percent seed transmission. This is likely the consequence of the major contribution of the percentage of seed transmission to the number of infected seeds. Indeed, equivalent models for *IS* constructed with the same estimators as those used for *ST* identified *VA_I_*, *SM*, and *V* as major determinants (not shown). These results highlight the complexity of the interactions between different infection traits in determining the contribution of vertical transmission to virus fitness.

In summary, by using a multivariate approach, this work provides a highly detailed analysis of the infection traits linked to the efficiency of plant virus seed transmission and identify virus within-host speed of movement and multiplication in the plant reproductive organs as major determinants of this process. We also show that a greater contribution of vertical transmission to virus fitness is associated with lower virus virulence. These results support theoretical predictions and contribute to shedding light on the mechanisms by which plant viruses achieve vertical transmission and optimize their fitness.

## MATERIALS AND METHODS

### *Arabidopsis* accessions and virus isolates.

Virus isolates UK1-TuMV (GenBank accession number AB194802), JPN1-TuMV (GenBank accession number KM094174), Fny-CMV (GenBank accession numbers NC_002034, NC_002035 and NC_001440), LS-CMV (GenBank accession numbers AF416899, AF416900, and AF127976), and De72-CMV (not sequenced) were used. JPN1-TuMV was obtained from a field-infected Raphanus sativus (Brassicaceae) plant (52), and De72-CMV was obtained from a field-infected Diplotaxis erucoides L. plant (Brassicaceae) (53); both viruses were propagated in Nicotiana benthamiana plants. UK1-TuMV, Fny-CMV, and LS-CMV were derived from biologically active clones (54–56) by *in vitro* transcription with T7 RNA polymerase (New England Biolabs, Ipswich, MA, USA), and transcripts were used to infect N. benthamiana plants for virus multiplication.

Eighteen *Arabidopsis* accessions were used (Table 1). Ten accessions represented the Eurasian geographic distribution of the species, and the remaining eight represented its distribution in the Iberian Peninsula, a Pleistocene glacial refuge for *Arabidopsis* (57). Plant seeds were stratified for 7 days at 4°C in pots with a diameter of 15 cm, in a 0.43-liter volume containing a 3:1 peat-vermiculite mix. Afterwards, pots were moved for seed germination and plant growth to a greenhouse at 22°C, under 16 h of light (intensity,120 to 150 mol s/m^2^). Plants were mechanically inoculated, either with N. benthamiana TuMV- and CMV-infected tissue ground in a solution containing 0.1 M Na_2_HPO_4_, 0.5 M NaH_2_PO_4_, and 0.02% DIECA (0.01 M phosphate buffer [pH 7.0], 0.2% sodium diethyldithiocarbamate) or with inoculation buffer for mock-inoculated plants. Inoculations were done when plants were at developmental stages 1.05 to 1.06 (58). After inoculation, all individuals were randomized in the greenhouse.

### Experimental design.

The 18 *Arabidopsis* accessions were inoculated with the 5 virus isolates as described above, with 10 replicates per treatment and accession. In these plants, virus multiplication, virulence, short-term seed survival, and efficiency of seed transmission were quantified as described below. Using these data, 6 out of the 18 *Arabidopsis* accessions were selected (An-1, Bay-0, Cad-0, Cum-0, Ll-0, and Fei-0) such that (i) accessions represented a range of TuMV and CMV seed transmissions (Fig. 1) and (ii) accessions with different life cycles were included (Table 1). We also selected one CMV isolate (Fny-CMV) and one TuMV isolate (JPN1-TuMV) for further experiments because they showed a wide range of percentages of seed transmission across accessions and were transmitted in a larger number of accessions.

Using the six *Arabidopsis* accessions and the two virus isolates, we conducted a time course experiment of viral infection. For each *Arabidopsis* accession, 85 plants per virus were inoculated with JPN1-TuMV and Fny-CMV each, and the other 10 were mock inoculated. Five infected plants per accession were harvested at regular intervals. Because each accession has a different developmental schedule (50, 51), intervals were established such that samples were collected from plant inoculation to silique ripening, and data from at least 15 time points were obtained. For each harvested plant, the amount of virus in the rosette and in 1-cm pieces of the inflorescence, which included inflorescence leaves, flowers, and siliques, if present, was quantified. These measures were used to calculate the speed of virus within-host movement. In parallel, 10 infected plants plus the mock-inoculated controls were allowed to complete their life cycle. In these plants, virus multiplication in the rosette and the inflorescence and rosette, inflorescence, and seed weights were obtained (see below). Seeds from these plants were used to estimate seed transmission rates and short-, medium-, and long-term seed survival. Note that the speed of virus within-host movement was measured through destructive sampling, whereas the other infection traits were quantified in the set of plants that completed their life cycle. Thus, for model building, the speed of virus within-host movement was considered an accession-specific trait (the averaged value derived from the destructive sampling was attributed to all plants of the same accession), whereas the other infection traits were considered plant-specific traits. Using this data set, we constructed global multivariate models that jointly considered all infection traits measured for both viruses as predictors of the efficiency of seed transmission as well as virus-specific models where infection traits were considered for each virus separately (see “Statistical analysis,” below). In this way, we could differentiate infection traits broadly associated with seed transmission from virus-specific determinants of this process.

To validate the accuracy of the constructed models, we went back to the set of 18 *Arabidopsis* accessions, retrieved the values of the parameters identified by our models as being relevant to predicting the efficiency of virus seed transmission, and interpolated these values in the constructed models. This allowed predicted values of seed transmission to be obtained, which were then compared with the values obtained experimentally. This approach allowed analysis of whether our models could be extrapolated to plant-virus interactions other than those utilized to build them and testing of their general accuracy.

### Virus multiplication.

TuMV and CMV multiplication was quantified as viral RNA accumulation via reverse transcription-quantitative PCR (qRT-PCR) for each individual plant. For plants included in the time course experiment, at each time point, virus accumulation in the rosette (*VA_R_*) was quantified from three disks with a diameter of 4 mm collected from different systemically infected leaves, and virus accumulation in the inflorescence (*VA_I_*) was quantified from the collected 1-cm pieces. For plants that were allowed to complete their life cycle, *VA_R_* and *VA_I_* were quantified at the end of the flowering period to ensure maximum viral multiplication in plant structures. Form these plant samples, total RNA extracts were obtained using TRIzol reagent (Life Technologies, Carlsbad, CA, USA), and 10 ng of total RNA was added to Brilliant III Ultra-Fast SYBR green qRT-PCR master mix (Agilent Technologies, Santa Clara, CA, USA) according to the manufacturer’s recommendations. Specific primers were used to amplify a 70-nucleotide (nt) fragment of the TuMV coat protein (CP) gene and a 106-nt fragment of the CMV CP gene (59, 60). Each plant sample was assayed in duplicate on a LightCycler 480 II real-time PCR system (Roche, Indianapolis, IN, USA). Absolute viral RNA accumulation was quantified as nanograms of viral RNA per microgram of total RNA, utilizing internal standards. For TuMV, internal standards consisted in 10-fold dilution series of plasmid-derived RNA transcripts of the same 70-nt CP fragment from UK1-TuMV. For CMV, 10-fold dilution series were prepared using purified viral RNA. All internal standards ranged from 2 × 10^−3^ ng to 2 × 10^−7^ ng.

### Virus speed of within-host movement.

The speed of virus within-plant movement (*SM*) in the plant inflorescence was quantified as centimeters per day from flower meristem formation to silique ripening, thus covering the time interval during which the virus can enter the seed. Following methods described previously (61, 62), at the time points defined in “Experimental design” above, the presence/absence of the virus in the 1-cm inflorescence pieces was monitored. Virus was detected via qRT-PCR as described above. As a result, we obtained a matrix of inflorescence height (in centimeters) versus time postbolting (days), in which we incorporated data on virus presence/absence for each height-time pair (see Table 3 in reference 62 for an example). The number of newly infected 1-cm segments (height) divided by the number of days that elapsed between two consecutive time points was used to calculate the speed of virus within-plant movement along the monitored period. Because virus speed of movement was analyzed for at least 15 time points, a minimum of 14 values were obtained. Virus speed of within-plant movement per accession was calculated by averaging these values between every two consecutive time points.

### Effect of infection on plant growth and reproduction.

Aboveground plant structures were harvested at complete senescence. The weights of the rosette (*RW*), inflorescence (*IW*), and seeds (*SW*) were obtained. *RW* was used as an estimate of plant resources dedicated to growth, and *IW* and *SW* were taken as estimates of plant resources dedicated to reproduction (63). The effect of virus infection on these traits was quantified by calculating ratios of infected to mock-inoculated plants for each of them, dividing the value of each infected plant by the mean value for the mock-inoculated plants of the same accession (Trait*_i_*/Trait*_m_*, where *i* and *m* denote infected and mock-inoculated plants, respectively). Virulence (*V*) was estimated as 1 minus the ratio of the total seed weight of infected (*SW_i_*) to the total seed weight of mock-inoculated (*SW_m_*) plants. The total numbers of seeds produced per mock-inoculated (*SN_m_*) and infected (*SN_i_*) plant were also quantified. To do so, we obtained the weight of 200 seeds for each replicate and derived the weight of a single seed. Using this value and the total seed weight of the corresponding plant, we calculated *SN*. Virus infection did not affect the weight of 200 seeds (*F* ≤ 2.79; *P* ≥ 0.114).

### Efficiency of virus seed transmission.

The efficiency of CMV and TuMV seed transmission was estimated both as a percentage of infected seeds and as the total number of infected seeds that gave rise to infected progeny per plant in grow-out tests. For each virus, 100 seeds per replicate were washed in a 10% bleach solution to ensure that any viral infection that occurred was not simply the result of the presence of virus on the seed coat but rather was the result of embryonic infection. Next, seeds were placed into petri dishes containing Murashige-Skoog medium, stratified for 3 days at 4°C, and germinated in a growth chamber at 22°C, under 16 h of light (intensity,120 to 150 mol s/m^2^). According to methods described previously (64), seedlings at 15 days poststratification were pooled in groups of 2 for a total of 50 groups per replicate. These groups were tested for the presence of TuMV or CMV via qRT-PCR as described above. Because we knew the proportion of samples that tested negative, we used a Poisson distribution to estimate the probability that more than one seedling would test positive in the same sample. The percentage of virus-infected seeds (*ST*) was then estimated using an expression reported previously (65), *p* = 1 − (1 − *y*/*n*)^1/^*^k^*, where *p* is the probability of virus transmission by a single seed, *y* is the number of positive samples, *n* is the total number of samples assayed (*n*  =  50), and *k* is the number of seedlings per sample (*k*  =  2). To calculate the total number of infected seeds per plant (*IS*), we multiplied *SN_i_* by *ST*.

### Seed survival.

We measured short-, medium-, and long-term seed survival as surrogates of the effect of infection on seed viability. Short-term seed survival was measured as percent germination of seeds derived from infected and mock-inoculated plants 4 months after harvest to avoid biases due to seed dormancy, according to the protocol described above. To estimate medium- and long-term seed survival, seeds were artificially aged, and their germination percentage was measured. The artificial aging process was a modification of the “basal thermotolerance assay” described previously (66). Briefly, seeds were incubated at 42°C for 24 h (medium term) or 48 h (long term) with a relative humidity of 100% and afterwards stratified for 3 days at 4°C. The germination percentage was measured every 24 h for 11 days, until a constant germination percentage was reached. Values of seed survival measures were derived from every replicate of each treatment and 100 seeds per replicate. Short-term (*G*_0_), medium-term (*G*_24_), and long-term (*G*_48_) seed survival were measured as the ratio of infected to mock-inoculated seed germination percentages. We quantified these three measures of seed survival because they can yield different information on the processes determining the efficiency of seed transmission: short-term survival occurs prior to our measures of seed transmission, and it can be considered a predictor of this trait. On the other hand, seed aging is posterior to our measures of seed transmission, and modifications of medium- and long-term survival can be considered a consequence of seed infection that modify the efficiency of seed transmission in the long run. In any case, the three seed survival measures can be considered potential estimators of virus efficiency of seed transmission.

### Statistical analysis.

Variables of virus seed transmission, virulence and the effect of virus infection on plant growth and reproduction, and seed survival were not normally distributed, and variances were heterogeneous according to Shapiro-Wilks and Levene tests, respectively. None of these variables could be normalized except for *SN_i_* and *IS*, which were normalized using a log transformation. Therefore, differences between virus species and plant accessions were analyzed by generalized linear models (GzLMs), applying the corresponding linked function and considering virus isolate nested to virus species and *Arabidopsis* accession as random factors. Virus species-specific analyses considered virus isolate and *Arabidopsis* accession as random factors (R package glmmTMB [67]).

The relationship between TuMV and CMV infection traits and the efficiency of seed transmission was analyzed by utilizing mixed-effect multiple-regression tests (68). We considered the following infection traits as potential estimators of the percentage of infected seeds: effect of the virus on plant rosette and inflorescence weights, virulence, speed of within-host movement, within-host multiplication in the rosette and inflorescence, and short-, medium-, and long-term seed survival. The same variables plus the percentage of infected seeds and the total number of seeds produced per plant were used as estimators of the total number of infected seeds. For model construction, the log transformation of the total number of seeds and of infected seeds per plant was used, which allowed scaling of all estimators. The cross-correlation between the estimators was analyzed using variance inflation factor (VIF). VIF values were lower than 3 for all variables, indicating minimal cross-correlation. Thus, all variables were included in the models. A set of models that included a global model containing all infection traits as fixed effects and nested models that contained all possible combinations of these traits were fitted for each response variable using general linear mixed models (R package glmmTMB [67]). The models combining data for TuMV and CMV included virus isolate and *Arabidopsis* accession as random effects, and TuMV- and CMV-specific models included only the latter random effect. Models for percent seed transmission were constructed using a Poisson function and a log-linked function, as this function best reflected the distribution of the data according to Akaike’s information criterion (AIC) (R package rriskDistributions [69]). Models for data on the number of infected seeds were constructed using a normal distribution and an identity-linked function. Global and nested models were ranked according to AIC scores, and the model with the lowest AIC score was selected as the best-ranked one. We calculated AIC Delta (Δ*_i_*) as the difference between the AIC of a given model and that of the best-ranked model (68). Finally, the Akaike relative weight (ω*_i_*) of each model was calculated according to the expression ω*_i_* = exp(−0.5Δ*_i_*)/Σexp(−0.5Δ*_i_*). The relative importance of a given estimator included in the best-ranked model was calculated by decomposing the *R*^2^ value of the model into components corresponding to each estimator using the R package relaimpo (70). Bivariate tests were used to analyze the association between the most relevant infection traits and the efficiency of virus seed transmission (R package stats [71]).

We analyzed the explanatory power of the best-ranked models using data derived from the 18 *Arabidopsis* accessions and the 5 virus isolates for which the efficiency of seed transmission was initially quantified. For this set of accessions, we quantified all the variables considered in the best-ranked models except for *SM*, *G*_24_, and *G*_48_. We estimated *SM* using values from the six-accession experiment. To do so, we calculated bivariate correlations between *SM* and all other variables. Because *SM* was an accession-specific trait, we used average values per accession. Based on these analyses, we identified the variable with the highest association with *SM*, and we used the equation of the linear relationship to estimate *SM* in the 18 *Arabidopsis* accessions. We could not follow the same approach for *G*_24_ and *G*_48_ as they were quantified for each individual plant, and no other trait significantly correlated with these two variables. Because these variables generally had very low relative importance in the best-ranked models, we considered them a constant in our simulations. *ST* and *IS* for the 18 *Arabidopsis* accessions were simulated using the best-ranked models (R package glmmTMB [67]), and their association with real values was analyzed by linear regressions.

## Supplementary Material

Supplemental file 1
